# 2D proteome analysis initiates new Insights on the *Salmonella *Typhimurium LuxS protein

**DOI:** 10.1186/1471-2180-9-198

**Published:** 2009-09-15

**Authors:** Gwendoline Kint, Kathleen AJ Sonck, Geert Schoofs, David De Coster, Jos Vanderleyden, Sigrid CJ De Keersmaecker

**Affiliations:** 1 Centre of Microbial and Plant Genetics, K. U. Leuven, Kasteelpark Arenberg 20, B-3001 Leuven, Belgium

## Abstract

**Background:**

Quorum sensing is a term describing a bacterial communication system mediated by the production and recognition of small signaling molecules. The LuxS enzyme, catalyzing the synthesis of AI-2, is conserved in a wide diversity of bacteria. AI-2 has therefore been suggested as an interspecies quorum sensing signal. To investigate the role of endogenous AI-2 in protein expression of the Gram-negative pathogen *Salmonella enterica *serovar Typhimurium (*S*. Typhimurium), we performed a 2D-DIGE proteomics experiment comparing total protein extract of wildtype *S*. Typhimurium with that of a *luxS *mutant, unable to produce AI-2.

**Results:**

Differential proteome analysis of wildtype *S*. Typhimurium versus a *luxS *mutant revealed relatively few changes beyond the known effect on phase 2 flagellin. However, two highly differentially expressed protein spots with similar molecular weight but differing isoelectric point, were identified as LuxS whereas the *S*. Typhimurium genome contains only one *luxS *gene. This observation was further explored and we show that the *S*. Typhimurium LuxS protein can undergo posttranslational modification at a catalytic cysteine residue. Additionally, by constructing LuxS-βla and LuxS-PhoA fusion proteins, we demonstrate that *S*. Typhimurium LuxS can substitute the cognate signal peptide sequences of β-lactamase and alkaline phosphatase for translocation across the cytoplasmic membrane in *S*. Typhimurium. This was further confirmed by fractionation of *S*. Typhimurium protein extracts, followed by Western blot analysis.

**Conclusion:**

2D-DIGE analysis of a *luxS *mutant *vs*. wildtype *Salmonella *Typhimurium did not reveal new insights into the role of AI-2/LuxS in *Salmonella *as only a small amount of proteins were differentially expressed. However, subsequent in depth analysis of the LuxS protein itself revealed two interesting features: posttranslational modification and potential translocation across the cytoplasmic membrane. As the *S*. Typhimurium LuxS protein does not contain obvious signal motifs, it is speculated that LuxS is a new member of so called moonlighting proteins. These observations might have consequences in future studies on AI-2 quorum signaling in *S*. Typhimurium.

## Background

Several bacteria utilize a cell-cell communication system called quorum sensing to coordinate diverse behaviors in response to population density [1]. This quorum sensing process is based on the generation of small signaling molecules by means of specific synthases. These signaling molecules accumulate into the extracellular environment and when a certain threshold concentration is reached, the bacteria detect and respond to this signal by altering their gene expression. Although several quorum sensing systems are known, the synthase highly conserved in many both Gram-negative and Gram-positive bacterial species is the quorum sensing synthase LuxS [2,3]. This enzyme catalyzes the conversion of *S-*ribosylhomocysteine to 4,5-dihydroxy-2,3-pentanedione (DPD) and homocysteine [4]. The unstable DPD spontaneously cyclizes into a family of interconverting molecules, collectively referred to as autoinducer-2 (AI-2) [5].

One of the first species reported to produce and respond to AI-2 resulting in expression of its luminescence genes is the marine pathogen *Vibrio harveyi *[6]. This bacterial species has been used as a bioreporter system in subsequent studies as it is able to detect and respond to AI-2 molecules produced by other bacteria that contain a functional LuxS protein [7,8]. AI-2 has therefore been postulated to be a universal language for interspecies communication. Based on the analysis of *luxS *mutants, a variety of phenotypes such as motility, cell division, virulence, biofilm formation, and bioluminescence have been attributed to AI-2 mediated quorum sensing [9,10]. However, the reaction catalyzed by LuxS is part of the activated methyl cycle, a metabolic pathway for the recycling of the major cellular methyl donor *S*-adenosylmethionine. As such, AI-2 can also be seen as a merely metabolic side product and the function of AI-2 might differ with the bacterial species under investigation [11]. In this respect it is interesting to note that in some cases, *luxS *phenotypes cannot be complemented by addition of exogenous AI-2 [12-16].

The only operon identified to date being directly regulated by AI-2 in *S*. Typhimurium, is the *lsr *operon encoding an ABC-type transporter for the uptake of AI-2 and some enzymes involved in AI-2 catabolism [17]. To date, the purpose of this uptake of AI-2 remains unclear. LuxS has also been linked to virulence, biofilm formation and flagellar phase variation [12,13,18,19]. For biofilm formation and flagellar phase variation, the phenotype could not be complemented by addition of synthetic DPD and consequently seem independent of AI-2 [12,13]. In order to get more insight in the role of AI-2 in *S*. Typhimurium, we performed a two-dimensional difference-in-gel electrophoresis experiment (2D-DIGE) comparing a *luxS *mutant with wildtype *S*. Typhimurium at the proteome level. Surprisingly, among the differential proteins identified, two distinct protein spots corresponded to LuxS. This observation was further explored and we show that in *S*. Typhimurium, LuxS can be posttranslationally modified on a cysteine residue that is crucial for enzymatic activity. Additionally, for the first time, evidence is presented that LuxS contains functional sequence information allowing translocation across the cytoplasmic membrane.

## Results

### 2D-DIGE analysis

**Figure 1:**
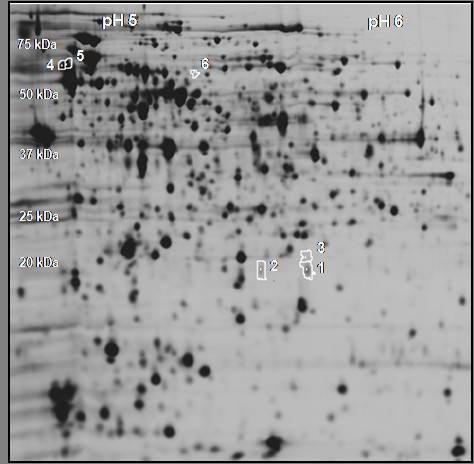
**Image of the master gel used in the 2D-DIGE analysis comparing the proteome of wildtype *S*. Typhimurium with that of a *luxS *mutant**. Spots with white spot boundaries were differentially expressed. The numbers indicated, correspond to the spot numbers in Table 1.

Total protein samples were taken from a wildtype *S*. Typhimurium strain and a *luxS *mutant. The mutant proteome was compared to that of the wildtype strain using 2D-DIGE. With this technique, protein samples are labelled prior to separation with up to three different fluorescent Cy dyes, allowing to load three different samples and incorporate an identical internal standard sample on each gel. Including such an internal standard, which is a pool of all experimental samples, minimizes the result variation related to the system, common in 2D-gelelectrophoresis (2DE) [20]. Details of the experimental setup can be found in the Methods section. Statistical analysis revealed 6 spots showing differential expression (p-value < 0.01 and fold increase/decrease > 1.5) between wildtype and the *luxS *mutant (see Figure 1). Differentially expressed protein spots were picked from preparative gels for identification with MALDI-TOF mass spectrometry (see Table 1). Due to low abundance, some spots could not be identified unambiguously, revealing a drawback of working with gel-based proteomics. Phase 2 flagellin was downregulated in the *luxS *mutant, corresponding to what was previously reported by Karavolos *et al*. [12]. An intriguing observation was the fact that two distinct protein spots, absent in the *luxS *mutant as compared to wildtype, were identified by mass spectrometry as being LuxS. This result led us to investigate the LuxS protein itself in more detail.

**Table 1:** Differentially expressed spots in the 2D-DIGE analysis

**Spot nr**.^**a**^	Name	Description	**Protein ID**^**b**^	**Av. Ratio**^**c**^	**p-value**^**d**^
*luxS *mutant *vs*. wildtype					

1	LuxS	*S*-ribosylhomocysteine lyase	Q9L4T0	-13.50	9.80E-04
2	LuxS	*S*-ribosylhomocysteine lyase	Q9L4T0	-9.77	1.70E-03
3	n.i.	n.i	n.i.	-3.94	7.00E-03
4	FljB	Phase 2 flagellin	P52616	-2.11	5.00E-04
5	FljB	Phase 2 flagellin	P52616	-1.75	8.00E-04
6	n.i.	n.i.	n.i.	-1.72	1.40E-03

^a ^Corresponding spot number on the gel image in Figure 1

^b ^Protein identification number

^c ^Average fold increase (positive ratio) or decrease (negative ratio) in expression of a protein in the mutant compared to the wildtype

^d ^P-value of the t-test analysis comparing the mutants to the wildtype

n.i. indicates not identified

### LuxS modification

**Figure 2:**
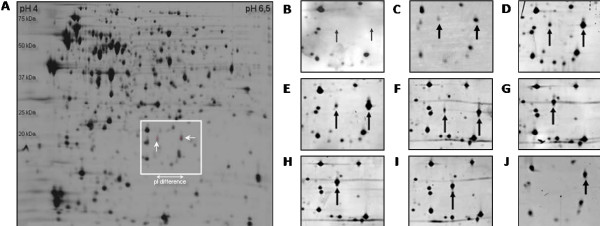
**2DE analysis of *Salmonella *Typhimurium *luxS *mutants**. (A) Total gel image of wildtype *S*. Typhimurium proteins. The two LuxS forms are indicated with an arrow. Based on pI calculations, the right spot corresponds to native LuxS and the left spot carries a posttranslational modification. (B-J) Close-up view of the area of the LuxS spots in a *luxS *mutant carrying different LuxS complementation constructs. (B) negative control - empty vector; (C) wildtype LuxS; (D) LuxS-Y88F; (E) LuxS-Y126F; (F) LuxS-Y131F; (G) LuxS-Y88D; (H) LuxS-Y126D; (I) LuxS-Y131D; (J) LuxS-C83A. Remark that in theory, on the gels from which panels G-I are taken, an additional modified LuxS spot is expected, accumulating the Y to D mutation and the cysteine modification.

Based on the relative position of the two LuxS spots on the gels and the theoretical pI of LuxS as calculated with ScanSite pI/MW, the most basic (right) spot (Figure 2A) corresponds to the native LuxS form while the other spot corresponds to LuxS with an additional negative charge. Efforts to identify the nature of this modification by tandem mass spectrometry were unsuccessful. Phosphorylation is a common posttranslational modification that induces a protein shift to the acidic side of 2D gels due to the negative charge of the phosphate group. Moreover, LuxS proteins from several Gram-negative bacteria contain a semi-conserved tyrosine phosphorylation site motif [21]. This led us to investigate whether the modification of LuxS in *S*. Typhimurium corresponds to a tyrosine phosphorylation. First, we attempted to detect a phosphorylated form of LuxS using the phosphospecific ProQ-Diamond stain (Invitrogen) on a 2D gel. However, no LuxS spot could be detected in this way (data not shown). Secondly, Western blotting using anti-phosphotyrosine antibodies was performed on an immunoprecipitated LuxS protein fraction. This immunoprecipitation step increases the LuxS concentration to facilitate detection of a putative phosphorylated form. Yet, LuxS could not be detected by these antibodies, making a tyrosine phosphorylation unlikely (data not shown). Subsequently, *luxS *constructs were made containing specific point mutations in each of the three LuxS tyrosine (Y) residues. These amino acids were changed into either a phenylalanine (F) residue that cannot become phosphorylated or an aspartate (D) residue to mimic a modification resulting in an additional negative charge. All constructs were functionally active, i.e. AI-2 was still produced by these modified proteins (data not shown). Total protein lysates of *S*. Typhimurium *luxS *mutant strains containing one of these point mutated LuxS constructs, were analyzed with 2D gel electrophoresis (2DE). As shown in Figure 2D-F, all strains with Y to F mutations still possess two LuxS spots. This rules out any of the tyrosine residues as target sites for modification. Furthermore, the pI shift seen in the Y to D mutation strains (Figure 2G-I) confirms the charge difference on the modified LuxS form. This result also illustrates that the interpretation of proteomic results has to be done with great care. Posttranslational modifications all correspond to a specific shift in pI and/or molecular weight. In this respect, we suggest that the postulated phosphorylation of LuxS in *Bifidobacterium longum *proposed by Yuan *et al*. should be re-investigated [22].

For *Bacillus subtilis *LuxS, oxidation of C84 has previously been reported with purified LuxS protein in studies to reveal the reaction mechanism of the synthase [23-25]. This oxidation is irreversible and adds one negative charge to the protein [23], which makes it a good candidate for the LuxS modification we detected in the *S*. Typhimurium proteome. Analogous to the tyrosine mutant constructs, we made a point mutation of the corresponding cysteine residue in *S*. Typhimurium to an alanine residue (C83A) which can no longer be oxidized and subsequently analyzed this strain by 2DE. As shown in Figure 2J the C83A *luxS *strain lacks the acid shifted LuxS spot confirming C83 as the target for posttranslational modification. As this cysteine residue is required for LuxS catalytic activity [26], the LuxSC83A mutant strain failed to produce AI-2 as revealed by the use of the AI-2 bioassay [27] (data not shown).

### LuxS localization

**Figure 3:**
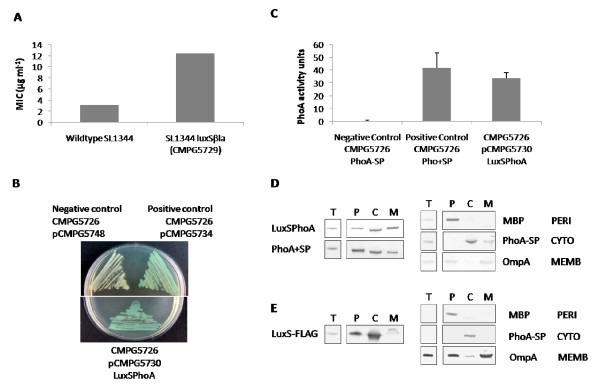
**Analysis of LuxS localization**. (A) Growth of *S*. Typhimurium wildtype and *luxSβla *with ampicillin. The minimal inhibitory concentration (MIC) for sensitivity to ampicillin (μg ml^-1^) in liquid culture was determined for each strain as described in the Methods section. These data are representative for three biological repeats. (B) Strains were grown on LB plates containing the chromogenic alkaline phosphatase substrate BCIP. Active alkaline phosphatase converts this substrate into a blue product. Negative and positive control strains express PhoA either without or with signal peptide (SP) from a constitutive promoter (pCMPG5748 and pCMPG5734); pCMPG5730 expresses a LuxS-PhoA fusion protein. All strains carry a Δ*phoN *mutation (CMPG5726). (C) Strains were grown to mid-exponential phase (OD_595 _1) and a PhoA activity test was performed. Average results of at least 3 biological replicates are shown with standard deviations. (D) Cellular fractionation of LuxS-PhoA fusion and control strains. (E) Cellular fractionation of *S*. Typhimurium expressing chromosomally FLAG-tagged LuxS. Total cells (T), grown to OD_595 _1, were separated into periplasmic (P), cytoplasmic (C) and membrane (M) fractions as described in the Methods section. The proteins maltose binding protein (MBP), alkaline phosphatase without signal peptide (PhoA-SP) and outer membrane protein A (OmpA) were used as periplasmic, cytoplasmic and membrane associated control proteins, respectively. All antibodies used are listed in the Methods section. Remarkably, in both panels D an E, the LuxS-PhoA fusion protein and FLAG-tagged LuxS protein respectively, seem to differ in molecular weight between the different fractions. This might be related to the unknown translocation mechanism.

Apart from posttranslational modification, we also examined the subcellular localization of LuxS, a feature that has not been investigated previously. It has been assumed that the LuxS protein localizes in the cytosol. A chromosomal translational fusion was made between LuxS and the periplasmic reporter protein β-lactamase. Expression of a β-lactamase results in resistance against β-lactam antibiotics such as ampicillin. However, to confer this resistance in Gram-negative bacteria, β-lactamase has to be exported outside the cytoplasm since formation of disulfide bridges is a prerequisite for enzyme activity [28,29]. An in frame gene construct encoding LuxS followed by a truncated β-lactamase lacking its native signal peptide was inserted into the chromosome of *S*. Typhimurium. The strain with the fusion construct was subsequently analyzed for growth at 37°C in liquid LB medium containing variable concentrations of ampicillin. As expected, a wildtype strain is highly sensitive to ampicillin. The *luxSβla *fusion strain, however, showed a clear increase in ampicillin resistance (Figure 3A). As the two strains differ also in synthesis of AI-2 because the LuxS-βla fusion protein is not expected to have AI-2 synthase activity, synthetic DPD was also added to the growth medium. However, this did not alter the observed difference in ampicillin resistance (data not shown). Increased ampicillin resistance and thus an active β-lactamase implies that the LuxS-βla fusion protein is translocated across the cytoplasmic membrane.

To confirm this interesting observation, a second fusion was made between LuxS and another periplasmic reporter protein, the alkaline phosphatase PhoA. Similar to β-lactamase, this enzyme requires disulfide bridge formation for correct folding and activity and has proven to be a useful tool for topology analysis [30]. An in frame gene construct encoding LuxS followed by a truncated PhoA lacking its native signal peptide was made. Additionally, two constructs encoding PhoA either with (positive control, PhoA+SP) or without (negative control, PhoA-SP) cognate signal peptide, both under the control of a constitutive promoter, were included in this experiment. To minimize background activity, a *Salmonella *Δ*phoN *strain lacking its own acid phosphatase gene was constructed and used for all further analyses. Results from the PhoA activity analysis are shown in Figure 3B-C. The strain with the *luxSphoA *fusion displays alkaline phosphatase activity similar to the positive control strain, both when grown on agar plates containing the chromogenic substrate 5-bromo-4-chloro-3-indolyl phosphate (BCIP) (Figure 3B) and in an enzymatic assay using *p*-nitrophenyl phosphate (pNPP) as a substrate (Figure 3C). Conversely, the negative control strain does not express active alkaline phosphatase, although the PhoA protein could be detected on a Western blot using anti-PhoA antibodies (Figure 3D), indicating that PhoA is present but remains in the cytoplasm in this negative control. Further direct proof for the subcellular location of the LuxS-PhoA fusion protein was obtained by subcellular fractionation of *S*. Typhimurium proteins into periplasmic, membrane and cytoplasmic fractions followed by Western blotting and detection with anti-PhoA antibodies. It can be seen that the LuxS-PhoA fusion protein is present in all fractions, similarly to the PhoA protein with its cognate signal peptide (PhoA+SP). The PhoA protein without its cognate signal peptide (PhoA-SP) is absent in the periplasmic fraction, as expected (Figure 3D). Detection of known control proteins (MBP for the periplasm and OmpA for the membrane fraction) shows that the fractionation protocol worked well, with only minor contaminations.

Finally, subcellular protein fractionation was performed on a *S*. Typhimurium strain chromosomally expressing C-terminal FLAG-tagged LuxS (CMPG5649). As shown in Figure 3E, the LuxS protein could be detected in all fractions though most abundant in the cytoplasmic fraction.

From the results of these three independent experimental approaches, it can be concluded that the *S*. Typhimurium LuxS protein must contain sequence information for membrane translocation. As *in silico *analysis with several publically available web tools such as PSORTb, LocTree and SignalP [31-33], did not reveal a typical Sec or Tat signal peptide for LuxS (data not shown), the mechanism for its membrane translocation remains unknown.

## Discussion

To further investigate the role of AI-2 in the pathogen *S*. Typhimurium, we evaluated a *luxS *mutant in a 2D-DIGE proteomics approach. Abolishment of AI-2 production does not cause a drastic change in the proteome of *S*. Typhimurium in our experimental set-up. Several factors should be kept in mind when interpreting this result. First, a proteome analysis is condition and time point dependent. Second, we used a 2D-DIGE approach to analyze the proteomic differences. The fluorescent labeling prior to protein separation permits the incorporation of an internal standard on each gel making differential proteome analysis more accurate [34]. In addition, we chose rather strict cut-off values in our statistical analysis to minimize false positive results. This specific experimental set-up could explain differences with a previously reported proteomic study on the effect of AI-2 in *Salmonella *[19]. Finally, the 2DE technique is limited both by the pI and molecular weight range of the first and second dimension, respectively, and by the low abundance of some protein spots which hampers their identification.

Nevertheless, 2DE is a powerful high-throughput technique revealing distinct posttranslational modified protein forms which are possibly relevant for the functionality of a protein. We identified two distinct protein forms of LuxS and this led us to examine this protein in more detail, more specifically considering posttranslational modification and subcellular localization. In previous publications it was already mentioned that the exact function and regulation of the LuxS protein, occurring in a wide diversity of bacteria, are probably more complex than anticipated so far [10,11,21,35]. However, apart from structural and catalytic studies, mainly in *B. subtilis*, the LuxS protein itself has not yet been subjected to further studies [23-26,36,37].

The two forms of the *S*. Typhimurium LuxS protein identified in this study have similar molecular weight, but differing isoelectric points. Point mutation analysis of the conserved cysteine 83 residue confirmed on the one hand its importance in the catalytic activity of *S*. Typhimurium LuxS and provided on the other hand clear evidence that the C83A mutation results in only one form of LuxS. From the latter observation, it can be concluded that the cysteine 83 residue is the subject of posttranslational modification of the wildtype LuxS protein in *S*. Typhimurium extending an observation previously reported for *Bacillus subtilis *[23-25]. This result shows that care has to be taken when interpreting putative posttranslational modifications. Although *S*. Typhimurium LuxS contains a semi-conserved tyrosine phosphorylation motif, our data do not support that tyrosine phosphorylation is involved.

The previous study of structure and catalytic mechanism of purified LuxS from the Gram-positive *B. subtilis *has demonstrated that a posttranslational modification can occur at the catalytic site [23-25]. In its active conformation, LuxS is a homodimer enclosing two identical active sites at the dimer interface each coordinating a Fe^2+ ^metal cofactor crucial for enzymatic activity [23]. Pei and coworkers suggest an oxidation mechanism similar to the one they described for peptide deformylase, another iron containing enzyme with the same coordinating amino acid residues as LuxS [23,38]. They hypothesize that cysteine modification is a consequence of the oxidation of the Fe^2+ ^ion coordinated within the active site of LuxS to Fe^3+ ^by molecular oxygen when substrate is unavailable. Consequently, Fe^3+ ^can no longer be coordinated within LuxS and leaves the protein.

Although the fate of LuxS lacking its iron cofactor and carrying an irreversible cysteine modification is currently unclear, this oxidation process could be a means of regulating the amount of active LuxS present in the cell according to the amount of substrate. AI-2 production has previously been linked to substrate availability in *S*. Typhimurium as *luxS *promoter activity has been shown to be constitutive under standard laboratory conditions [39]. It will be of interest to further investigate the link between substrate availability and posttranslational modification of LuxS.

Another feature of LuxS in *S*. Typhimurium, namely its subcellular localization, was studied using complementary approaches. Our results indicate that LuxS can be translocated across the plasma membrane. This could explain the observation of Agudo *et al*., who identified LuxS in an overall screening as differentially expressed in the periplasmic protein fraction of a *S*. Typhi *dsbA *mutant lacking a major disulfide isomerase enzyme [40]. In bacteria, two major translocase systems are known to date, i.e. the Sec and Tat pathway [41]. However, extensive *in silico *analysis of the *S*. Typhimurium LuxS protein did not reveal a typical Sec or Tat signal peptide for LuxS translocation. Future wet lab experiments involving *Salmonella *Sec and Tat mutants are required to elaborate further on this.

LuxS is not the first enzyme for which an unexpected localization is observed. An increasing number of both prokaryotic and eukaryotic proteins are being found in cellular compartments in addition to the compartment where their function is best described. They are referred as promiscuous or moonlighting proteins [42,43]. Having multiple locations within the cell is a typical feature of some moonlighting proteins that can contribute to a functional switch. These functions can be enzymatic, but even structural or regulatory functions are common. Moreover, many moonlighting proteins are conserved in evolution, a feature of LuxS [3].

Given the more likely cytoplasmic location of the known substrate of LuxS, *S*-ribosyl homocysteine, we propose a dual, meaning at both sides of the cytoplasmic membrane, localization for LuxS. It will be of interest to further refine experiments of localization in view of our observation that a significant band can be observed in the membrane fraction. However, at this stage, we can only hypothesize what the functional implications of the extracytoplasmic location of LuxS, as revealed in this study, could be. A kind of shuttling mechanism between cytoplasm and periplasm might occur to regulate the amount of active LuxS. This might be linked to a posttranslational modification occurring outside the cytoplasmic space when substrate is unavailable.

## Conclusion

A 2D-DIGE experiment comparing a *luxS *mutant, unable to synthesize the quorum sensing signal AI-2, with wildtype *S*. Typhimurium did not reveal many differences on the proteome level. Nevertheless, two separate forms of LuxS with similar molecular weights but differing isoelectric points were identified. Based on this result, we focused specifically on LuxS. Here, we show that in *S*. Typhimurium, LuxS is partly posttranslationally modified involving a conserved cysteine residue and occurs at both sides of the cytoplasmic membrane. This research emphasizes the strength of high-throughput gel-based proteome analysis for getting new insights in posttranslational protein regulation. At this stage we do not know whether membrane translocation and posttranslational modification are coupled and how these processes are related to AI-2 signaling. Nevertheless, these insights feed challenging research on LuxS-based quorum sensing in *S*. Typhimurium and possibly even other bacterial species.

## Methods

### Bacterial strains and growth conditions

All strains and plasmids that were used in this study are listed in Table 2. *Salmonella *Typhimurium SL1344 is the wildtype strain [44]. For the 2D-DIGE analysis, *Salmonella *strains were grown under *in vivo *mimicking conditions. Growth monitoring during 48 h revealed that all strains grow very much alike under the conditions tested. The *luxS *mutant is unable to produce AI-2 due to the lack of a crucial enzyme in the AI-2 synthesis pathway. An overnight preculture in 5 ml Luria-Bertani broth (LB) medium supplemented with 0.5% glucose was diluted 1:100 in 100 ml LB medium with 0.5% glucose, flushed with a gas mixture of 97% N_2 _and 3% O_2 _during 15 minutes prior to inoculation and sealed air-tight with a rubber cap to mimic the low oxygen concentration known to induce expression of *Salmonella *invasion proteins [45]. The cultures were incubated non-shaking at 37°C for 5 h. In all validation experiments, *Salmonella *strains were grown with aeration at 37°C in Luria-Bertani broth (LB) medium [46]. Antibiotics were applied at the following concentrations: 25 μg/ml chloramphenicol (for plasmids based on pAYC184) and 100 μg/ml ampicillin (for plasmids based on pFAJ1708). For the determination of the MIC of ampicillin, variable concentrations of ampicillin were used (serial diluted twofold from 100 μg ml^-1 ^to 3.125 μg ml^-1^) [47]. Synthetic DPD (Omm Scientific Inc.) and BCIP (Sigma) were used in concentrations of 70 μM and 50 μg ml^-1^, respectively. Standard protocols were used for molecular cloning [46]. All primers used are listed in Table 3. All strains and constructs were finally verified by PCR and sequencing analysis. The *S*. Typhimurium SL1344 *phoN *mutant (CMPG5726), the *luxSβla *fusion mutant (CMPG5729), and the FLAG-tagged LuxS strain (CMPG5649) were all constructed with the procedure described by Datsenko and Wanner [48] starting from the plasmids pKD4 [48], pTn5-blam [49] and pSUB11 [50], respectively. The *luxS *mutant was constructed by cloning the *luxS *gene and flanking regions from SL1344 into the *Sac*I restriction site of plasmid pUCBM20 (Boehringer-Mannheim) using primers PRO-196 and PRO-197, resulting in plasmid pCMPG5700. A kanamycin resistance cassette was amplified from pUC18-2 [51] using primers PRO-194 and PRO-195, containing *Cla*I restriction sites. Subsequently, this cassette was inserted into the *Cla*I restriction site of the *luxS *gene. The mutated *luxS *gene was then cloned into the *Sac*I restriction site of suicide plasmid pCVD442 [52]. A chromosomal *luxS *mutant of SL1344 was obtained by double homologous recombination.

**Table 2:** Bacterial strains and plasmids

Name	Description	Reference
*S*. Typhimurium SL1344	Parent strain	[44]
CMPG5649	*S*. Typhimurium *luxS3xFLAG*	This work
CMPG5702	Insertion of a kanamycin resistance gene in the *ClaI *restriction site of the *luxS *gene of SL1344;*luxS*::*Km*^*R*^	This work
CMPG5726	*S*. Typhimurium SL1344 Δ*phoN*	This work
CMPG5729	*S*. Typhimurium SL1344 *luxSβla*	This work
pTn5-blam	Plasmid used as template for construction of CMPG5729	[49]
pKD4	Plasmid used as template for construction of CMPG5726	[48]
pSUB11	Plasmid used as template for construction of CMPG5649	[50]
pCVD442	Positive selection suicide vector	[52]
pACYC184	Derivative of p15A; Tc^R^; Cm^R^	[54]
pFAJ1708	Derivative of RK-2; Ap^R^; Tc^R^; contains *npt*II promoter of pUC18-2	[55]
pCMPG5664	pACYC184 containing the *luxS *gene of SL1344	[13]
pCMPG5700	pUCBM20 containing the *luxS *gene and flanking regions of SL1344 in *Sac*I restriction site; Ap^R^, *lacZ*	This work
pCMPG5718	pACYC184 with *luxS*: Y_88_→F_88_	This work
pCMPG5719	pACYC184 with *luxS*: Y_88_→D_88_	This work
pCMPG5720	pACYC184 with *luxS*: Y_126_→F_126_	This work
pCMPG5721	pACYC184 with *luxS*: Y_126_→D_126_	This work
pCMPG5722	pACYC184 with *luxS*: Y_131_→D_131_	This work
pCMPG5723	pACYC184 with *luxS*: Y_131_→F_131_	This work
pCMPG5730	pACYC184 vector containing *luxSphoA *fusion protein with *luxS *promoter	This work
pCMPG5734	pFAJ1708 containing PhoA with signal peptide; positive control	This work
pCMPG5748	pFAJ1708 containing PhoA without signal peptide; negative control	This work
pCMPG5743	pCMPG5664 with point mutation in *luxS*: C_83_→A_83_	This work

**Table 3:** Primers used in this study

Primer	**Sequence**^**a**^	**Purpose**^**b**^
PRO-194	AAATCGATAGGTCGACGGGCCCGGTACC	FW CMPG5702
PRO-195	AAATCGATCGCTGCCGCAAGCACTCAGG	RV CMPG5702
PRO-196	AAGAGCTCCATGTACTACCTGCCGTATGCG	FW pCMPG5700
PRO-197	AAGAGCTCACGTATCCTGATTCAGCGGG	RV pCMPG5700
PRO-510	GCCGCACCGGCTTTT**T**CATGAGCCTGATTGG	FW Y_88_→F_88_
PRO-511	CCAATCAGGCTCATG**A**AAAAGCCGGTGCGGC	RV Y_88_→F_88_
PRO-512	GCCGCACCGGCTTT**G**ACATGAGCCTGATTGG	FW Y_88_→D_88_
PRO-513	CCAATCAGGCTCATGT**C**AAAGCCGGTGCGGC	RV Y_88_→D_88_
PRO-514	GGAGCTGAACGTTT**T**CCAGTGCGGTACG	FW Y_126_→F_126_
PRO-515	CGTACCGCACTGG**A**AAACGTTCAGCTCC	RV Y_126_→F_126_
PRO-516	GGAGCTGAACGTT**G**ACCAGTGCGGTACG	FW Y_126_→D_126_
PRO-517	CGTACCGCACTGGT**C**AACGTTCAGCTCC	RV Y_126_→D_126_
PRO-518	CCAGTGCGGTACGT**T**TCAGATGCACTCGC	FW Y_131_→F_131_
PRO-519	GCGAGTGCATCTGA**A**ACGTACCGGACTGG	RV Y_131_→F_131_
PRO-520	CCAGTGCGGTACG**G**ATCAGATGCACTCGC	FW Y_131_→D_131_
PRO-521	GCGAGTGCATCTGAT**C**CGTACCGGACTGG	RV Y_131_→D_131_
PRO-0177	TTGCACTTCCTTTCATTTGCTGTGGCCAGTTTGCGGGAAGACTTTCACCTGTGTAGGCTGGAGCTGCTTC	FW CMPG5726
PRO-0178	CATTATAGGATTACATCTGTTTATTATTGCCTGATCCGGAGTGAGTCTTTCATATGAATATCCTCCTTA	RV CMPG5726
PRO-1428	AGCTGGCGCTGCCGAAAGAAAAACTGCAGGAACTGCATATTCTGTCTCTTATACACATCTCA	FW CMPG5729
PRO-1429	TAAACCGGGGTTAATTTAAATACTGGAACCGCTTACAAATAAGAGTCTCTTATACACATCTGGT	RV CMPG5729
PRO-0889	CTCGCCGATGGGC**GC**CCGCACCGGCTTTTAC	FW C_83_→A_83_
PRO-0890	GTAAAAGCCGGTGCGG**GC**GCCCATCGGCGAG	RV C_83_→A_83_
PRO-208	ATGAATTCGCGGCACCGGGAAAGCGTTCGG	FW *luxSphoA *fusion
PRO-0415	GTTTCCAAGCTTATATGCAGTTCCTGCA	RV *luxSphoA *fusion
PRO-0719	GAAGGGTCTAGATGAAACAAAGCACTA	FW PhoA with signal peptide
PRO-1273	ATTCTAGACATGGAGAAAATAAAATGCCTGTTCTGGAAAACCG	FW PhoA without signal peptide, contains ribosome binding site
PRO-0721	ATCTGCAGTTATTTCAGCCCCAGAG	RV PhoA control plasmids
PRO-0238	GCTGGCGCTGCCGAAAGAAAAACTGCAGGAACTGCATATTGACTACAAAGACCATGACGG	FW CMPG5649
PRO-0239	CCGGGGTTAATTTAAATACTGGAACCGCTTACAAATAAGACCATATGAATATCCTCCTTAG	RV CMPG5649

^a ^Point mutations are indicated in bold

^b ^FW: Forward primer; RV: Reverse primer

### Construction of LuxS point mutation vectors

Point mutations were introduced into the coding sequence of *luxS*, in order to change the different tyrosine (Y) residues in phenylalanine (F) or aspartate (D). To this end, the QuickChange site-directed mutagenesis kit (Stratagene, USA) was used. Shortly, point mutations were introduced by PCR in plasmid pCMPG5700, carrying the *luxS *gene and flanking regions, using the different primers listed in Table 3. After restriction digesting the mother strand DNA with *Dpn*I, competent *E. coli *DH5α cells were transformed with the mutated plasmid DNA. Transformants were selected on LB containing 100 μg/ml ampicillin, 50 μg/ml IPTG and 40 μg/ml X-gal. Constructs were verified by sequencing. Subsequently, the mutated *luxS *genes (*Bam*HI-*Eco*47III fragments) were cloned into the *Bam*HI and *Eco*RV restriction sites of vector pACYC184, which is stable in *Salmonella*. The resulting plasmids were electroporated into CMPG5702.

pCMPG5743 containing a C83A point mutation in *luxS *was constructed based on pCMPG5664 - expressing *luxS *from its own promoter - according to the Quick site-directed mutagenesis protocol (Stratagene, USA) and sequenced to confirm the point mutation.

### Construction of PhoA constructs

For the construction of the *luxSphoA *fusion construct (pCMPG5730), *S*. Typhimurium SL1344 *luxS *was amplified by PCR using PRO-208 and PRO-0415. The *luxS *fragment was cloned into a pCRIITOPO vector (Invitrogen) and subsequently subcloned in the *Hin*dIII site of the PhoA fusion vector pPHO7 [53], kindly provided by Prof. C. Gutierrez. Finally, the LuxS-PhoA fusion protein under control of the *luxS *promoter was subcloned as a blunt ended *Ecl*136II fragment into the *Eco*RV site of a *Salmonella *compatible pACYC184 vector [54]. Positive and negative PhoA control constructs (pCMPG5734 and pCMPG5748) were made by cloning the PhoA coding sequence with or without signal peptide, amplified by PCR with PRO-0719/PRO-1273 and PRO-0721, into the *Xba*I and *Pst*I cloning site of pFAJ1708, an RK-2 derived low-copy-number expression vector containing the *nptII *promoter of pUC18-2 [55]. All constructs were verified by PCR and sequencing and finally electroporated to the CMPG5726 background. For protein fractionation analysis of FLAG-tagged LuxS, the negative PhoA control construct pCMPG5748 was electroporated to the CMPG5649 background and used as cytoplasmic control protein.

### Determination of β-lactamase minimal inhibitory concentrations

The minimal inhibitory concentrations (MIC) were determined as previously described [47].

### PhoA activity assay

Alkaline phosphatase assays were performed according to the procedure of Daniels *et al*. [56].

### 2D gel electrophoresis

Total protein sampling and 2D-DIGE analysis were essentially performed as previously described [57]. Four biological replicates were taken for each strain of which two were labeled with Cy3 and two were labeled with Cy5. The internal standard sample was labeled with Cy2 and included on each gel, while the other protein samples were randomized across all gels. The first dimension was performed on 24 cm Immobiline DryStrips with a 3-7 non-linear pH range (GE Healthcare). Analysis of the gel images was performed using DeCyder™ 6.5 software (GE Healthcare). A t-test analysis was used to identify spots that were differentially expressed between the two strains. Spots with a p-value < 0.01 and a more than 1.5 fold change in expression level were considered differentially expressed. For identification, spots of interest were manually matched to the protein pattern in the preparative gel images and included in a pick list. Spot picking was executed automatically with the Ettan SpotPicker (GE Healthcare). For 2DE analysis of LuxS point mutant strains, protein samples were taken at OD_595 _1 and 30 μg protein was loaded per strip. Gels were stained with Sypro Ruby (Invitrogen).

### Cell fractionation and Western blotting

Cells were grown in LB medium to mid-exponential phase (OD_595 _1). Total protein samples were taken as described by Sittka *et al*. [58]. For SDS-PAGE, 0.01 OD was loaded. Cell fractionation was performed according to a procedure from Randall *et al*. [59]. Periplasmic, cytoplasmic and membrane protein fractions were quantitated with the *RC DC *protein assay from Bio-rad and 10 μg was loaded per lane.

The antibodies used included mouse monoclonal anti-PhoA (Sigma, 1:10 000) for detection of the LuxS-PhoA fusion protein; mouse polyclonal anti-PhoA (Chemicon Int., 1:5000) for detection of PhoA expressed by the control plasmids; rabbit anti-MBP (New England Biolabs, 1:5000); rabbit anti-OmpA [60]; goat anti-mouse alkaline phosphatase IgG (Sigma, 1:10 000) and goat anti-rabbit alkaline phosphatase IgG (Sigma, 1:10 000).

## Abbreviations

2DE: two-dimensional gel electrophoresis; 2D-DIGE: two-dimensional difference-in-gel electrophoresis; AI-2: autoinducer-2; βla: β-lactamase; BCIP: 5-bromo-4-chloro-3-indolyl phosphate; DPD: 4,5-dihydroxy-2,3-pentanedione; MALDI-TOF: matrix-assisted laser desorption/ionization - time of flight; MW: molecular weight; pI: isoelectric point; pNPP: *p*-nitrophenyl phosphate

## Authors' contributions

GK designed and performed the study, and drafted the manuscript. KAJS participated in the design of the study and performed the 2D-DIGE analysis and analysis of the posttranslational modification. GS participated in the 2DE analysis of point mutants. DDC carried out part of the molecular cloning work and Western blotting. JV and SCJDK conceived the study, participated in its design and coordination and helped to draft the manuscript. All authors read and approved the final manuscript.
